# *Candida auris*, what do paediatricians need to know?

**DOI:** 10.1136/archdischild-2017-313960

**Published:** 2018-03-15

**Authors:** Adilia Warris

**Keywords:** candida auris, candida haemulonii, neonates, children, multidrug resistance

## Abstract

The newly recognised and emerging fungal species, *Candida auris*, has caused worldwide invasive infections and has been implicated in difficult to control hospital outbreaks. Challenges are encountered in the correct identification of this fungus as commonly used phenotypic and biochemical methods fail to differentiate *C. auris* from other *Candida* species. Its resistance profile, over 90% of isolates are fluconazole resistant and 35% are resistant to amphotericin, confronts clinicians with the restricted arsenal of antifungals and concerns about optimal treatment. The very first *C. auris* isolate was recovered from a paediatric patient in retrospect. Although infections with the more antifungal-resistant *Candida* species are less frequently observed in paediatric patients, this seems to be different for *C. auris* infections.

## Introduction

The emergence of *Candida auris* has received worldwide attention, not only from the scientific and clinical communities, but has as well been picked up by several public media channels. *C. auris* being reported as a ‘sometimes deadly and often resistant fungal infection’, ‘a superbug highly resistant to traditional drugs’ and ‘*C. auris* sickens dozens’, makes clear that there is something to be feared from this newly recognised *Candida* species.

The yeast *C. auris* was described for the first time in 2009 on recovery from the external ear canal of a Japanese patient.^1^ Subsequently, *C. auris* was recognised as causing chronic otitis media in 15 patients in Korea, including three paediatric patients.^2^ The earliest case to date was identified in retrospect by DNA sequencing of a Korean bloodstream isolate from a paediatric surgery patient in 1996.^3^ Since 2009, patients colonised and infected with *C. auris* have been reported from nearly all continents (except for Australia and Antarctica). *C. auris* has been shown to be a challenge to identify and treat, is capable to cause difficult to manage hospital outbreaks and reasons for its emergence is far from clear.^4 5^

The vast majority of infections caused by *Candida* species in the paediatric population are caused by *C. albicans* or *C. parapsilosis*, while the more antifungal resistant species as *C. glabrata* and *C. krusei* are less prevalent in comparison to *Candida* infections in adult patients.^6 7^ As the very first reports of *C. auris* infections are described in both neonates and children as well as adults, attention needs to be paid to its consequences for the management of *Candida* infections in the paediatric population.

## Clinical epidemiology

A literature search, restricted to publications in English, including publications up to 8 December 2017, was performed by using Medline/Pubmed. At that moment, published clinical reports of *C. auris* infections originated from Japan, South Korea, India, Kuwait, Oman, South Africa, Venezuela, Panama, Colombia, Pakistan, Israel, Spain, UK, Canada and the USA describing 109 patients with candidaemia, 19 patients with chronic otitis media or otitis externa and 28 patients with other infections (including candiduria/urinary tract infections (n=16) and wound and soft tissue infections (n=6)).^1–4 8–21^ Real-time data from the Centers for Disease Control and Prevention show that new cases are reported on an almost weekly basis with a total of 243 clinical cases of *C. auris* infection in the USA.^22^ Public Health England shows a more episodic pattern in reported clinical cases (56 in total) with high increases during outbreaks.^23^ No paediatric patients have been reported in the UK (Dr E M Johnson, Public Health England, personal communication).

Ten out of the 18 publications described infections in adult patients only. Two publications did include paediatric patients but no details were given separately for the patients <18 years of age^19 21^ or age was not mentioned.^13^ Twenty-three paediatric patients have been reported in five case series published, of which 20 neonates and children suffered from a blood stream infection (18% of the total population).^3 8 9 12^ Three other paediatric patients suffered from chronic otitis media with positive ear swabs for *C. auris*.^2^ Table 1 summarises the clinical characteristics of those infections. Paediatric patients have only been reported in Asia and South America. Underlying conditions and risk factors are comparable to those known to render paediatric patients at risk for developing candidaemia and invasive candidiasis. Of the 20 paediatric patients with candidaemia due to *C. auris*, 14 were neonates and/or born prematurely. Older infants and children developed *C. auris* candidaemia during intensive care unit (ICU) admissions, postsurgery or with an underlying haematological malignancy. Antifungal regimens prescribed varied hugely with half of the patients receiving combination antifungal therapy with two or three antifungals. Mortality was 30% in paediatric patients with *C. auris* blood stream infections and lower compared with adult patients with mortality rates ranging from 30% to 60%. In contrast, during the *C. auris* outbreak in a large UK hospital, no attributable deaths were observed due to *C. auris* infections.^4^

**Table 1:** Clinical epidemiology of published paediatric cases with *Candida auris* infections

Patient number (reference)	Country	Sex/age	Underlying condition	Positive cultures	Localisation of infection	Treatment	Outcome
1 (Lee *et al* 3)	South Korea	F/1 year	Traumatic respiratory arrest Aspiration pneumonia	Blood	Blood stream	FLU, removal CVC	Survived
2 (Chowdhary *et al* 8)	India	F/3 days	Prematuritas, TEF, ICH, bacterial sepsis	Blood	Blood stream	Caspo	Died
3 (Chowdhary *et al* 8)	India	F/10 days	Prematuritas, ELBW, bacterial sepsis	Blood	Blood stream	d-AmB	Survived
4 (Chowdhary *et al* 8)	India	F/28 days	Pneumonia, late-onset sepsis	Blood	Blood stream	d-AmB	Survived
5 (Chowdhary *et al* 8)	India	F/45 days	Meningitis, septic shock, cardiac defect	Blood	Blood stream	d-AmB	Survived
6 (Chowdhary *et al* 8)	India	M/10 years	ALL, chronic kidney disease	Blood	Blood stream	none	Died
7 (Chowdhary *et al* 9)	India	M/2 years	Short-bowel syndrome, intestinal perforation, pneumonia, bacterial sepsis	Blood, tip CVC	Blood stream	FLU, d-AmB, removal CVC	Survived
8 (Calvo *et al* 12)	Venezuela	F/30 days	Prematuritas, sepsis	Blood	Blood stream	AmB, VORI	Died
9 (Calvo *et al* 12	Venezuela	F/13 days	Prematuritas, colon atresia, sepsis, abdominal surgery	Blood	Blood stream	VORI, AmB, Caspo	Died
10 (Calvo *et al* 12)	Venezuela	M/17 days	Abdominal surgery, sepsis	Blood	Blood stream	FLU	Died
11 (Calvo *et al* 12)	Venezuela	F/23 days	Prematuritas, septic shock	Blood	Blood stream	Caspo	Survived
12 (Calvo *et al* 12)	Venezuela	M/18 days	Prematuritas, sepsis	Blood	Blood stream	FLU, VORI, Caspo	Died
13 (Calvo *et al* 12)	Venezuela	M/2 days	Intestinal atresia, congenital heart disease, abdominal surgery	Blood	Blood stream	Caspo	Survived
14 (Calvo *et al* 12)	Venezuela	M/12 days	NEC, HIE, sepsis, abdominal surgery	Blood	Blood stream	VORI, Caspo	Survived
15 (Calvo *et al* 12)	Venezuela	M/11 days	Prematuritas, NEC, sepsis, abdominal surgery	Blood	Blood stream	AmB, Caspo	Survived
16 (Calvo *et al* 12)	Venezuela	F/18 days	Prematuritas, abdominal wall defect, sepsis, abdominal surgery	Blood	Blood stream	VORI	Survived
17 (Calvo *et al* 12)	Venezuela	F/14 years	Septic shock	Blood	Blood stream	VORI, Anidula, Caspo	Survived
18 (Calvo *et al* 12)	Venezuela	F/10 days	Prematuritas, HIE, sepsis	Blood	Blood stream	FLU	Survived
19 (Calvo *et al* 12)	Venezuela	F/2 months	Meningocele, congenital hydrocephalus, sepsis	Blood	Blood stream	VORI, Caspo	Survived
20 (Calvo *et al* 12)	Venezuela	M/1 month	Prematuritas, RDS, sepsis	Blood	Blood stream	VORI, Caspo	Survived
21 (Kim *et al* 2)	Korea	Not available	Chronic otitis media	Ear	Ear	n.a.	Survived
22 (Kim *et al* 2)	Korea	Not available	Chronic otitis media	Ear	Ear	n.a.	Survived
23 (Kim *et al* 2)	Korea	Not available	Chronic otitis media	Ear	Ear	n.a.	Survived

ALL, acute lymphoblastic leukaemia; AmB, amphotericin B; anidula, anidulafungin; caspo, caspofungin; CVC, central vascular catheter; d-AMB, amphotericin deoxycholate; ELBW, extreme low-birth weight; HIE, hypoxic ischaemic encephalopathy; ICH, intracerebral haemorrhage; FLU, fluconazole; NEC, necrotising enterocolitis; RDS, respiratory distress syndrome; TEF, trachea o esophageal fistula; VORI, voriconazole.

As *C. auris* has been commonly misidentified as *Candida haemulonii*, a rare encountered *Candida* species causing invasive infections.^3 8 10 11 13^ Paediatric patients with *C. haemulonii* blood stream infections reported in the literature have been summarised in table 2. The clinical characteristics are comparable to those described for *C. auris* fungaemia and neonates and children with well-known underlying conditions and risk factors to develop invasive fungal disease are affected. Thirteen paediatric patients from Kuwait, Korea and Brazil have been described, including five premature neonates.^2 24–26^ *C. haemulonii* infections have been more often described in infants and children (62%) compared with *C. auris* infections affecting mostly neonates (70%). Half of the patients with *C. haemulonii* infections were treated with combination antifungal therapy and the mortality rate was 30%.

**Table 2:** Clinical epidemiology of published paediatric cases with fungaemia caused by *Candida haemulonii* and *Candida pseudohaemulonii*

Patient number (reference)	Country	Sex/age	Underlying condition	Candida species	Treatment	Outcome
1 (Khan *et al* 24)	Kuwait	M/6 weeks	Prematuritas, hernia diaphragmatica	*C. haemulonii*	L-AmB, Caspo, FLU	Died
2 (Khan *et al* 24)	Kuwait	M/10 weeks	Prematuritas, ELBW	*C. haemulonii*	FLU	Survived
3 (Khan *et al* 24)	Kuwait	F/7 weeks	Prematuritas, ELBW	*C. haemulonii*	d-AmB, Caspo	Survived
4 (Khan *et al* 24)	Kuwait	F/1 month	Prematuritas, LBW	*C. haemulonii*	L-AmB	Died
5 (Kim *et al* 2)	Korea	M/10 years	Nasopharyngeal cancer, upper gastrointestinal bleeding	*C. haemulonii*	ITRA, d-AmB, FLU	Survived
6 (Kim *et al* 2)	Korea	M/10 months	Necrotising enterocolitis	*C. pseudohaemulonii*	FLU	Survived
7 (Kim *et al* 2)	Korea	M/2 years	Acquired combined immunodeficiency	*C. pseudohaemulonii*	d-AmB	Died
8 (Kim *et al* 2)	Korea	F/2 months	Necrotising enterocolitis	*C. pseudohaemulonii*	FLU, d-AmB, L-AmB	Survived
9 (Kim *et al* 2)	Korea	F/12 months	Cardiac defect	*C. pseudohaemulonii*	L-AmB, FLU	Died
10 (Kim *et al* 2)	Korea	F/2 years	Cardiac defect	*C. pseudohaemulonii*	FLU	Survived
11 (Muro *et al* 25)	Brazil	F/19 months	Down syndrome, cardiac surgery, chylothorax	*C. haemulonii*	d-AmB, L-AmB, FLU	Survived
12 (Muro *et al* 25)	Brazil	F/9 years	Ewing’s sarcoma, febrile neutropaenia	*C. haemulonii*	d-AmB	Survived
13 (Silva *et al* 26)	Brazil	F/3 weeks	Prematuritas, ELBW	*C. haemulonii*	FLU	Survived

Caspo, caspofungin; d-AMB, amphotericin deoxycholate; ELBW, extreme low birth weight; FLU, fluconazole; L-AmB, liposomal amphotericin B; LBW, low birth weight; ITRA, itraconazole.

Due to the low numbers of paediatric patients described, it is difficult to draw meaningful comparisons with *Candida* infections caused by other species, specific patient groups affected, geographic patterns and the outcome of the infections.

## Identification and susceptibility

*C. auris* isolates have been misidentified as a range of other *Candida* species by using conventional phenotypic and biochemical methods. Most commonly, these isolates have been misidentified as *C. haemulonii*, as *C. auris* is phylogenetically closely related to the *C. haemulonii* species complex.^3 8 10 11 13^ *C. haemulonii* complex species are less frequently detected than *C. auris*, although inaccuracies with the molecular identification of less common *Candida* species prevents a robust insight into these infections.^5^ It is also possible that some of the reported isolates of *C. haemulonii* are misidentified as *C. auris*. It has been suggested that chromogenic agar is a low-cost method to differentiate between *C. auris* and *C. haemulonii* isolates.^5^ Molecular techniques are recommended to identify *C. auris* to the species level. Matrix-assisted laser desorption ionisation–time of flight mass spectrometry is capable of providing an accurate identification to the species level once spectra for *C. auris* are present in its database.^5^ The development of *C. auris-*specific PCR assays allows for rapid identification and are of particular use during outbreak settings.^5^ Sequencing of genetic loci and internal transcribed spacer domains of the rRNA provides the ability to differentiate between geographic clades.^5 21^ Genome sequencing has been shown to be useful in the identification of *C. auris* but is often not an available technique in most laboratories and is less feasible for routine identification purposes.

Intrinsic susceptibility patterns for *C. auris* show elevated minimum inhibitory concentrations for all three classes of antifungals, the polyenes, the azoles and the echinocandins.^27^ Exact breakpoints as have been established for the common *Candida* species using the Clinical and Laboratory Standards Institute and the European Committee on Antimicrobial Susceptibility Testing methodologies have not been established.^28^ Antifungal susceptibility data for 54 *C. auris* isolates showed that 93% were resistant to fluconazole (≥32 mg/L), 54% to voriconazole (≥2 mg/L), 35% to amphotericin B (≥2 mg/L) and 7% to echinocandins (≥8 mg/L).^3^ Forty-one per cent of those isolates were resistant to greater than or equal to two classes of antifungals. Resistance to all three classes of antifungals was observed in two Indian isolates.^21^ Of the 13 *C. haemulonii* infections in paediatric patients, eight isolates (62%) showed amphotericin B resistance, two isolates (15%) showed resistance for fluconazole and two (15%) for echinocandins.^2 24–26^

## Emergence of *C. auris*

Results from whole-genome sequencing studies suggest that *C. auris* has emerged near simultaneously in four or more locations rather than spreading from a single source.^21^ Sequencing of *C. auris* isolates in the UK have clearly shown that those isolates have several geographic origins and belong to at least three different clades.^29^ This suggest that *C. auris* isolates have been introduced into the UK from different locations.

But what has driven the emergence of this new *Candida* species? One of the explanations could be that *C. auris* has just not been recognised before, but this is not supported by retrospective reviews of large collections of *Candida* isolates. A thorough review of over 15 000 *Candida* isolates from four continents (SENTRY isolate collection) did not detect any *C. auris* isolate before 2009.^21^ Increasing antifungal selection pressures either in humans and/or animals and/or the environment may cause the emergence of a new multidrug-resistant *Candida* species. The availability of antifungals with an improved toxicity profile has contributed to an expansion in the prescriptions of antifungals for prophylactic and empirical use. Increased antifungal use in agriculture and in the clinical environment has led to well-recognised emerging resistance among *Aspergillus fumigatus* (azole resistance) and *C. glabrata* (echinocandin resistance).^30^ However, selection by antifungal pressure alone seems to be less likely as non-*albicans Candida* species have been increasing since the introduction of fluconazole in 1990. Changes to the ecological niches of *C. auris* and intrinsic characteristics as numerous virulence attributes, thermotolerance and salt tolerance and aggregation into difficult-to-disperse clusters, may have allowed the emergence of this newly recognised species causing human infections and persistence in hospital environments.^31^ A number of molecular resistance mechanisms are well described for causing resistance to particular antifungals in several *Candida* species.^28^ As yet, molecular mechanisms resulting in antifungal resistance in *C. auris* need to be elucidated.

## Clinical management

Based on susceptibility data, echinocandins seem to be the drug of choice as less than 10% resistance is reported.^27 28^ Micafungin showed increased efficacy in a murine study of *C. auris* candidaemia compared with fluconazole and amphotericin B.^32^ For the paediatric population, micafungin and caspofungin can be used while awaiting susceptibility testing results.^33 34^ Depending on the site of infection, alternative choices might be considered.^5 28 33 34^ Echinocandins have limited penetration in the cerebrospinal fluid and urine, and therefore other antifungal compounds should be used to treat central nervous system or renal tract infections.^35^ No recommendations can be made with regard to treatment with combinations of antifungals, as data are not available.

Consideration should be given to determine *Candida* isolates from mucosal surfaces and non-sterile sites to the species level and perform susceptibility testing. Particularly, in paediatric patients at high risk for developing invasive *Candida* infections, as these results may guide early and targeted treatment if a *Candida* infection is suspected based on clinical signs and symptoms. Even more so in premature neonates where a high percentage of false-negative blood cultures for *Candida* species has been described.^36^

An additional reason to be informed about colonising *C. auris* isolates in intensive care settings is to prevent transmission and to instal proper infection prevention and control measures. Several countries including the UK have released guidance documents with recommendations regarding screening policies, isolation of patients, contact precautions and cleaning of equipment and clinical environments.^5 21^

## Summary

*C. auris* infections have emerged as an important challenge in the management of already vulnerable paediatric patients including premature neonates, infants and children admitted to ICUs and those with underlying malignancies. Proper identification is challenging with conventional methodologies failing. Great concern exists how to control this emerging new *Candida* species showing multidrug resistance and being very well capable to persist in hospital environments. The increased number of cases detected worldwide is causing a global concern and research is urgently needed. Current research is addressing the many unanswered questions related to the emergence of this *Candida* species, its molecular resistance and virulence mechanisms and improved management options to minimise its impact on patient outcomes.

## References

[1] Satoh K, Makimura K, Hasumi Y, et al Candida auris sp. nov., a novel ascomycetous yeast isolated from the external ear canal of an inpatient in a Japanese hospital. Microbiol Immunol 2009;53:41–4. 10.1111/j.1348-0421.2008.00083.x 19161556

[2] Kim MN, Shin JH, Sung H, et al Candida haemulonii and closely related species at 5 university hospitals in Korea: identification, antifungal susceptibility, and clinical features. Clin Infect Dis 2009;48:e57–61. 10.1086/597108 19193113

[3] Lee WG, Shin JH, Uh Y, et al First three reported cases of nosocomial fungemia caused by Candida auris. J Clin Microbiol 2011;49:3139–42. 10.1128/JCM.00319-11 21715586 PMC3165631

[4] Schelenz S, Hagen F, Rhodes JL, et al First hospital outbreak of the globally emerging Candida auris in a European hospital. Antimicrob Resistance Infect Control 2016;5:35 10.1186/s13756-016-0132-5 PMC5069812 27777756

[5] Jeffery-Smith A, Taori SK, Schelenz S, et al Candida auris: a review of the literature. Clin Microbiol Rev 2018;31:e00029–17. 10.1128/CMR.00029-17 29142078 PMC5740969

[6] Arendrup MC, Dzajic E, Jensen RH, et al Epidemiological changes with potential implication for antifungal prescription recommendations for fungaemia: data from a nationwide fungaemia surveillance programme. Clin Microbiol Infect 2013;19:e343–53. 10.1111/1469-0691.12212 23607326

[7] Palazzi DL, Arrieta A, Castagnola E, et al Candida speciation, antifungal treatment and adverse events in pediatric invasive candidiasis: results from 441 infections in a prospective, multi-national study. Pediatr Infect Dis J 2014;33:1294–6. 10.1097/INF.0000000000000431 24892850

[8] Chowdhary A, Sharma C, Duggal S, et al New clonal strain of Candida auris, Delhi, India. Emerg Infect Dis 2013;19:1670–3. 10.3201/eid1910.130393 24048006 PMC3810747

[9] Chowdhary A, Anil Kumar V, Sharma C, et al Multidrug-resistant endemic clonal strain of Candida auris in India. Eur J Clin Microbiol Infect Dis 2014;33:919–26. 10.1007/s10096-013-2027-1 24357342

[10] Magobo RE, Corcoran C, Seetharam S, et al Candida auris-associated candidemia, South Africa. Emerg Infect Dis 2014;20:1250–1. 10.3201/eid2007.131765 24963796 PMC4073876

[11] Emara M, Ahmad S, Khan Z, et al Candida auris candidemia in Kuwait, 2014. Emerg Infect Dis 2015;21:1091–2. 10.3201/eid2106.150270 25989098 PMC4451886

[12] Calvo B, Melo AS, Perozo-Mena A, et al First report of Candida auris in America: clinical and microbiological aspects of 18 episodes of candidemia. J Infect 2016;73:369–74. 10.1016/j.jinf.2016.07.008 27452195

[13] Vallabhaneni S, Kallen A, Tsay S, et al Investigation of the first seven reported cases of candida auris, a globally emerging invasive, multidrug-resistant fungus – United States, May 2013–August 2016. Am J Transplant 2017;17:296–9. 10.1111/ajt.14121 28029734

[14] Ruiz Gaitán AC, Moret A, López Hontangas JL, et al Nosocomial fungemia by Candida auris: first four reported cases in continental Europe. Rev Iberoam Micol 2017;34:23–7. 10.1016/j.riam.2016.11.002 28131716

[15] Al-Siyabi T, Al Busaidi I, Balkhair A, et al First report of Candida auris in Oman: clinical and microbiological description of five candidemia cases. J Infect 2017;75:373–6. 10.1016/j.jinf.2017.05.016 28579303

[16] Mohsin J, Hagen F, Al-Balushi ZAM, et al The first cases of Candida auris candidaemia in Oman. Mycoses 2017;60:569–75. 10.1111/myc.12647 28685887

[17] Arauz AB, Caceres DH, Santiago E, et al Isolation of Candida auris from 7 patients in Central America: importance of accurate diagnosis and susceptibility testing. Mycoses 2017:1–4. 10.1111/myc.12709 28945325

[18] Ben-Ami R, Berman J, Novikov A, et al Multidrug-resistant Candida haemulonii and C. auris, Tel Aviv, Israel. Emerg Infect Dis 2017;23:195–203. 10.3201/eid2302.161486 PMC5324804 28098529

[19] Morales-López SE, Parra-Giraldo CM, Ceballos-Garzón A, et al Invasive infections with multidrug-resistant yeast Candida auris, Colombia. Emerg Infect Dis 2017;23:162–4. 10.3201/eid2301.161497 27983941 PMC5176232

[20] Schwartz IS, Hammond GW First reported case of multidrug-resistant Candida auris in Canada. Can Commun Dis Rep 2017;43:150–3. 10.14745/ccdr.v43i78a02 29770082 PMC5764715

[21] Lockhart SR, Etienne KA, Vallabhaneni S, et al Simultaneous emergence of multidrug-resistant Candida auris on 3 continents confirmed by whole-genome sequencing and epidemiological analyses. Clin Infect Dis 2017;64:134–40. 10.1093/cid/ciw691 27988485 PMC5215215

[22] www.cdc.gov/fungal/diseases/candidiasis/candida-auris.html (accessed on 22 Feb 2018)

[23] www.gov.uk/government/publications/candida-auris-emergence-in-england (accessed on 22 Feb 2018).

[24] Khan ZU, Al-Sweih NA, Ahmad S, et al Outbreak of fungemia among neonates caused by Candida haemulonii resistant to amphotericin B, itraconazole, and fluconazole. J Clin Microbiol 2007;45:2025–7. 10.1128/JCM.00222-07 17428940 PMC1933024

[25] Muro MD, Motta FA, Burger M, et al Echinocandin resistance in two Candida haemulonii isolates from pediatric patients. J Clin Microbiol 2012;50:3783–5. 10.1128/JCM.01136-12 22895037 PMC3486200

[26] Silva CM, Carvalho-Parahym AM, Macêdo DP, et al Neonatal candidemia caused by Candida haemulonii: case report and review of literature. Mycopathologia 2015;180:69–73. 10.1007/s11046-015-9872-7 25666389

[27] Arendrup MC, Prakash A, Meletiadis J, et al Comparison of EUCAST and CLSI reference microdilution MICs of eight antifungal compounds for Candida auris and associated tentative epidemiological cutoff values. Antimicrob Agents Chemother 2017;61:e00485–17. 10.1128/AAC.00485-17 28416539 PMC5444165

[28] Arendrup MC, Patterson TF Multidrug-resistant Candida: epidemiology, molecular mechanisms, and treatment. J Infect Dis 2017;216:S445–51. 10.1093/infdis/jix131 28911043

[29] Borman AM, Szekely A, Johnson EM Isolates of the emerging pathogen Candida auris present in the UK have several geographic origins. Med Mycol 2017;55:563–7. 10.1093/mmy/myw147 28204557

[30] Perlin DS, Rautemaa-Richardson R, Alastruey-Izquierdo A The global problem of antifungal resistance: prevalence, mechanisms, and management. Lancet Infect Dis 2017;17:e383–92. 10.1016/S1473-3099(17)30316-X 28774698

[31] Clancy CJ, Nguyen MH Emergence of Candida auris: an international call to arms. Clin Infect Dis 2017;64:141–3. 10.1093/cid/ciw696 27989986

[32] Lepak AJ, Zhao M, Berkow EL, et al Pharmacodynamic optimization for treatment of invasive Candida auris Infection. Antimicrob Agents Chemother 2017;61:e00791–17. 10.1128/AAC.00791-17 28584152 PMC5527602

[33] Hope WW, Castagnola E, Groll AH, et al ESCMID* guideline for the diagnosis and management of Candida diseases 2012: prevention and management of invasive infections in neonates and children caused by Candida spp. Clin Microbiol Infect 2012;18(Suppl 7):38–52. 10.1111/1469-0691.12040 23137136

[34] Pappas PG, Kauffman CA, Andes DR, et al Executive summary: clinical practice guideline for the management of Candidiasis: 2016 update by the infectious diseases society of America. Clin Infect Dis 2016;62:409–17. 10.1093/cid/civ1194 26810419

[35] Felton T, Troke PF, Hope WW Tissue penetration of antifungal agents. Clin Microbiol Rev 2014;27:68–88. 10.1128/CMR.00046-13 24396137 PMC3910906

[36] Warris A, Lehrnbecher T Progress in the diagnosis of invasive fungal disease in children. Curr Fungal Infect Rep 2017;11:35–44. 10.1007/s12281-017-0274-9 28680525 PMC5487864

